# Pupillary Dilation in an Adolescent With Psychogenic Non-epileptic Seizures: A Case Report

**DOI:** 10.7759/cureus.106007

**Published:** 2026-03-27

**Authors:** Joyce S Li, Faraz A Khan, Omar A Danoun

**Affiliations:** 1 Psychiatry, Wayne State University School of Medicine, Detroit, USA; 2 Child and Adolescent Psychiatry, Henry Ford Health System, Detroit, USA; 3 Neurology, Henry Ford Health System, Detroit, USA; 4 Neurology and Ophthalmology, Michigan State University, East Lansing, USA; 5 Palestinian Neuroscience Initiative, Al-Quds University, Abu Dis, PSE

**Keywords:** autonomic nervous system dysfunction, case report, child and adolescent psychiatry, functional neurological disorder, psychogenic non epileptic seizure, psychological trauma, pupillary dilation

## Abstract

Psychogenic non-epileptic seizures (PNES) are functional episodes that mimic epileptic seizures without epileptiform activity. We describe a 16-year-old female with trauma history, mood disorder, and a recent suicide attempt who developed recurrent PNES during hospitalization. Episodes featured unresponsiveness, irregular shaking, and heightened sympathetic arousal with tachycardia, hypertension, diaphoresis, and pupillary dilation up to 8 mm. Events were often prolonged or clustered and triggered by environmental stressors. Given concerns for epileptic seizures, the patient underwent medical evaluation, which revealed no epileptiform activity captured on video-electroencephalogram during events and was consistent with a diagnosis of PNES. Management included monitoring, sertraline titration, and behavioral strategies such as mindfulness techniques and minimizing unnecessary staff interventions during episodes. By discharge, the patient no longer endorsed suicidal ideation; had shown a reduction in frequency and severity of PNES episodes; and was motivated to continue care. This case highlights pupillary dilation as an underrecognized finding in PNES and underscores the need for further study to clarify its prevalence, mechanisms, and diagnostic implications.

## Introduction

Psychogenic non-epileptic seizures (PNES) are paroxysmal events characterized by seizure-like behaviors, such as generalized shaking or sudden falls, that resemble epileptic seizures but occur in the absence of epileptiform activity on electroencephalography [1,2]. PNES arise from a multifactorial biopsychosocial etiology involving maladaptive stress responses, trauma exposure, psychiatric comorbidities, and dysregulation of limbic-motor and autonomic networks, resulting in abnormal integration of emotional and physiological processes rather than epileptogenic pathology [1-4]. They account for 20-30% of referrals to tertiary epilepsy centers and commonly co-occur in patients with a history of epilepsy, making PNES a frequent and challenging diagnostic dilemma [1-3]. PNES are further associated with significant morbidity, misdiagnosis, and unnecessary healthcare utilization, including inappropriate anti-epileptic drug use, repeated emergency visits, intensive care admissions, and a 2.5-fold higher rate of all-cause mortality from the general population [2-6]. This rate is comparable to that seen in drug-resistant epilepsy and is largely driven by external causes such as suicide and substance- or medication-related poisoning [6]. Given the substantial clinical overlap between PNES and epileptic seizures, considerable effort has been directed toward identifying clinical features that distinguish the two conditions [1,3,4].

In general, epileptic seizures are often associated with stereotyped motor activity, ictal incontinence, lateral tongue biting, and postictal confusion, whereas PNES more commonly features asynchronous or thrashing movements, eye closure with preserved pupillary light reflex, and prolonged event duration [1-3,7]. The gold standard for diagnosing PNES is demonstrating the absence of epileptiform activity on video-electroencephalography (vEEG) during a typical event in conjunction with clinical features suggestive of a non-epileptic etiology [1,2,4]. Accurate diagnosis requires integration of seizure semiology, vEEG findings, and clinical history [2-4]. Beyond motor semiology, autonomic dysregulation is increasingly recognized in PNES, including alterations in heart rate variability (HRV) and preictal autonomic dynamics, even among patients with comorbid epilepsy [8-11]. Despite these differences, no single pathognomonic sign reliably distinguishes epileptic seizures from PNES, underscoring the importance of recognizing overlapping features, including pupillary dilation, which remains infrequently documented despite evidence of occurrence in both conditions [3].

Changes in pupil size reflect autonomic nervous system (ANS) activity in response to psychological and emotional stimuli and have been implicated in mental health conditions as a physiological index of autonomic functioning [12,13]. Pupillary dilation (mydriasis), defined as enlargement of the pupil beyond its normal resting diameter due to increased sympathetic or decreased parasympathetic tone, has been noted in early descriptive reports of psychogenic seizures and was typically attributed to stress-related sympathetic activation [14,15]. However, these observations have not been systematically characterized and lack diagnostic specificity. Contemporary literature distinguishing PNES from epileptic seizures frequently emphasizes the preservation of the pupillary light reflex in PNES [1,2,4]; yet, pupillary dilation itself is rarely addressed as a shared autonomic manifestation. In the present case, the patient demonstrated bilateral pupillary dilation to approximately 8 mm on multiple occasions, with equal, reactive light responses, consistent with transient mydriasis. Given that pupillary dilation is more commonly associated with epileptic seizures and infrequently discussed in PNES, this finding increased concern for epileptic seizure activity and prompted escalation of care. We present this case to highlight pupillary dilation as a potential shared feature between psychogenic and epileptic seizures, one that remains infrequently documented in contemporary literature. Understanding such overlapping autonomic features may help prevent misinterpretation of bedside findings when evaluating seizure-like events.

## Case presentation

A 16-year-old female with a history of obesity, major depressive disorder (MDD), anxiety, chronic self-injurious behavior (SIB), and PNES was admitted to our behavioral health hospital following a suicide attempt by intentional overdose. Three months earlier, she developed seizure-like episodes after reporting head trauma during a physical altercation with a biological parent. PNES was subsequently confirmed at an outside hospital using vEEG and neuroimaging (reports unavailable). She was later removed from parental custody and experienced multiple disrupted foster placements due to worsening psychiatric symptoms, including passive suicidal ideation (SI) and escalating SIB, as well as frequent PNES episodes. Before a scheduled psychiatric evaluation could occur, she ingested 15 tablets of lorazepam (1 mg each) along with unknown quantities of sertraline, ibuprofen, and acetaminophen. She later disclosed a recent sexual assault by a known adult male involving oral and vaginal penetration, which she identified as precipitating the suicide attempt.

In the ED, she was tachycardic but hemodynamically stable and in no acute distress. Her psychiatric history included MDD characterized by low and irritable mood, insomnia, reduced appetite, and passive SI, as well as unspecified anxiety with excessive worry. She reported a five-year history of cutting behaviors. Home medications included sertraline 25 mg daily (taken inconsistently) and a one-time prescription for lorazepam 1 mg as needed for PNES episodes; she denied prior regular benzodiazepine use and reported ingesting the entire supply during the attempt. Laboratory evaluation demonstrated mild leukocytosis, microcytic anemia, hypomagnesemia, borderline hypokalemia, and a mildly elevated anion gap (Table 1). Urine toxicology was positive for benzodiazepines and cannabinoids. She was medically stabilized with electrolyte repletion and cardiac monitoring before transfer for inpatient psychiatric care.

**Table 1:** Selected laboratory findings during ED evaluation These findings reflect mild electrolyte abnormalities and toxicology results obtained during the patient’s ED evaluation. Only abnormal or clinically relevant laboratory results are shown. Values are reported with corresponding reference ranges. WBC: white blood cell count, RDW: red cell distribution width

Test	Result	Reference range
WBC	13.23 ×10³/µL	4.50–12.00
Hemoglobin	10.8 g/dL	11.5–16.0
RDW	15.90%	11.5–14.5
Magnesium	1.7 mg/dL	2.1–2.8
Potassium	3.5 mmol/L	3.5–5.5
Anion gap	15.5 mmol/L	4.0–12.0
Benzodiazepine screen (urine)	Positive	Negative
Cannabinoid screen (urine)	Positive	Negative

Developmental history was notable for premature birth with intrauterine crack cocaine exposure and neonatal respiratory complications requiring supplemental oxygen during the first year of life. Socially, she was a high school student, identified as a cisgender heterosexual female, and reported no history of romantic or sexual relationships. She was a temporary ward of the state residing with a friend’s parent pending placement, with ongoing legal proceedings involving her biological parents. She had one adult biological sibling and multiple half-siblings, including two younger siblings placed in separate foster homes. She denied regular alcohol or illicit drug use, reporting only a single recent marijuana exposure. Family history was notable for maternal bipolar disorder, schizophrenia in the maternal grandmother, and paternal alcohol-use disorder.

On psychiatric intake, she appeared guarded, with slow speech and a constricted affect, but denied active SI. Superficial linear scars were noted on the bilateral upper and lower extremities. Insight and judgment were assessed as poor, given her limited appreciation of illness severity and demonstrated impulsivity. The remainder of her mental status examination was unremarkable.

**Figure 1:**
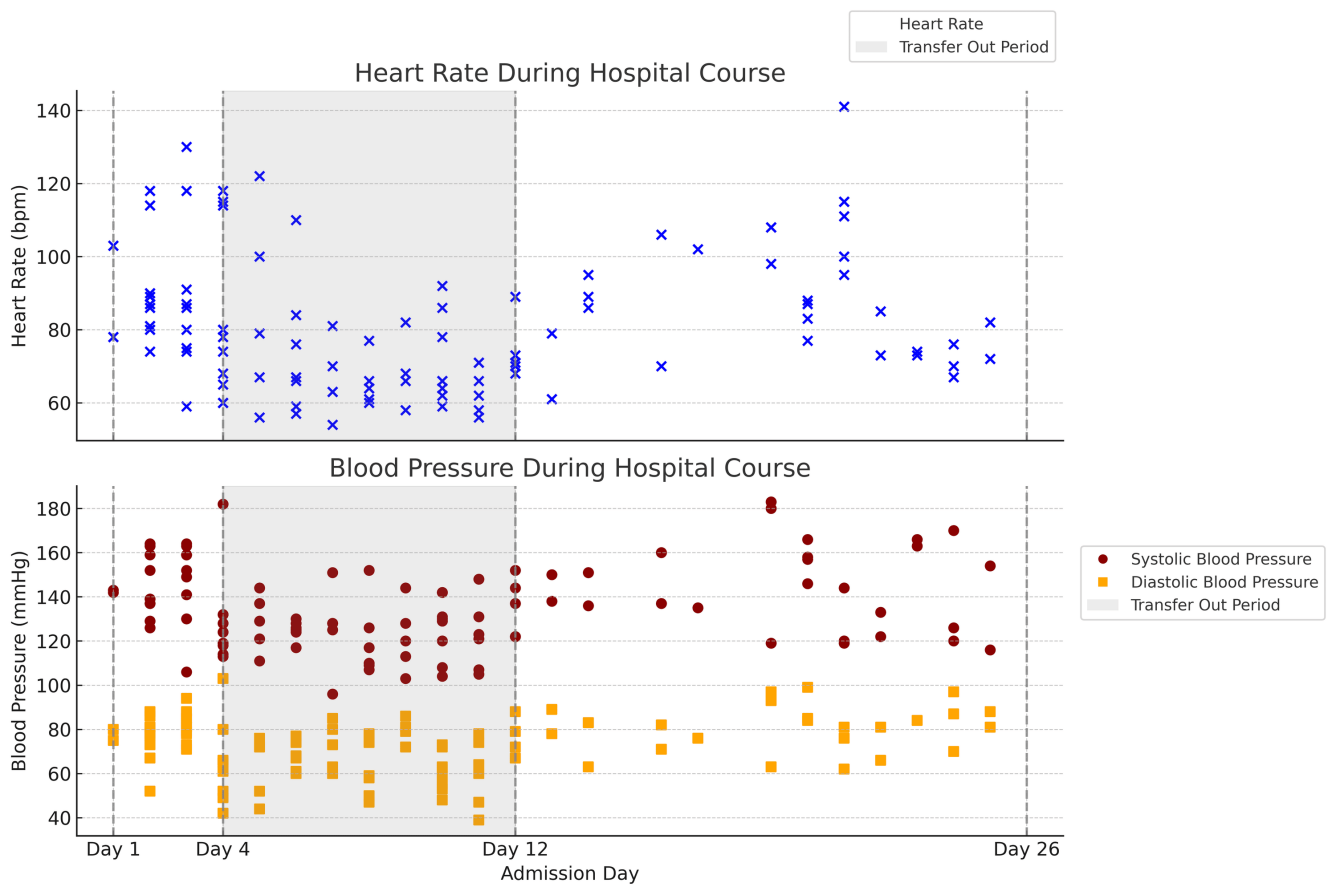
Scatterplots of heart rate (top) and blood pressure (bottom) readings obtained during PNES episodes over the hospital course These vital-sign changes illustrate episodic autonomic activation during PNES events. Heart rate values are plotted as blue “X” markers, systolic pressures as red circles, and diastolic pressures as yellow squares. Vertical dashed lines mark the patient’s transitions between hospital settings. The gray-shaded area indicates when the patient was transferred for medical evaluation. Apart from the gray area, which includes all recorded vitals, readings were obtained only during PNES episodes. PNES: psychogenic non-epileptic seizure Image Credit: Authors using Python (Matplotlib, Pandas; Python Software Foundation, Wilmington, DE, USA)

During admission, she experienced recurrent PNES episodes characterized by unresponsiveness, large-amplitude asynchronous limb movements, head shaking, and autonomic activation, including tachycardia, hypertension, clamminess, and pupillary dilation up to 8 mm with preserved reactivity (Figure 1). Vocalizations, vomiting, and drooling sometimes accompanied episodes, but there was no tongue biting, urinary incontinence, or airway compromise. Events occurred multiple times daily in clusters lasting approximately 5-10 minutes, followed by rapid return to baseline orientation and vital signs, with diffuse body pain and fatigue. The pre-admission frequency of PNES episodes was not documented, although collateral reports described “significant activity” similar to her inpatient presentation. Observed episodes with dilated yet reactive pupils were also documented in nursing notes during hospitalization.

**Figure 2:**
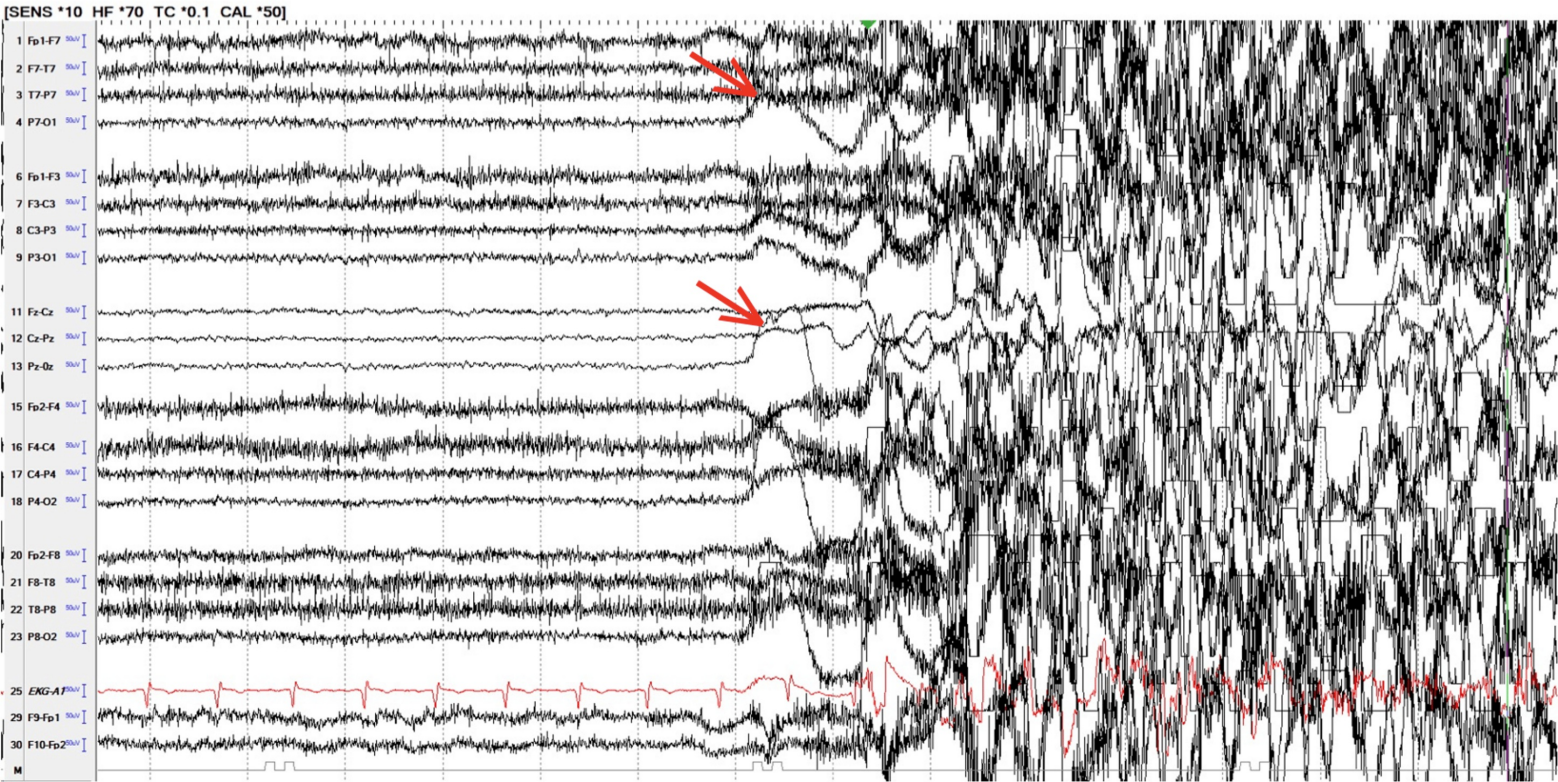
EEG recording of a PNES event The initial half of the EEG demonstrates a normal background rhythm during a period of eye closure, motionlessness, and unresponsiveness. The latter half shows diffuse movement artifacts corresponding to whole-body shaking. The red arrows indicate a few examples of such artifacts. The presence of a normal background rhythm and absence of epileptiform activity support a diagnosis of PNES. EEG: electroencephalography, PNES: psychogenic non-epileptic seizure Image Credit: Authors using Python (Matplotlib, Pandas; Python Software Foundation, Wilmington, DE, USA)

One event was notable for prolonged duration (~45 minutes), escalating motor activity, vomiting, and sustained autonomic changes. Given concern for a possible epileptic seizure, lorazepam 2 mg was administered intramuscularly at approximately 30 minutes, and emergency medical services were activated. Following transfer to the medical facility, vEEG captured multiple typical events without epileptiform activity (Figure 2), confirming PNES. Brain MRI was unremarkable, and she returned for continued inpatient psychiatric management.

**Figure 3:**
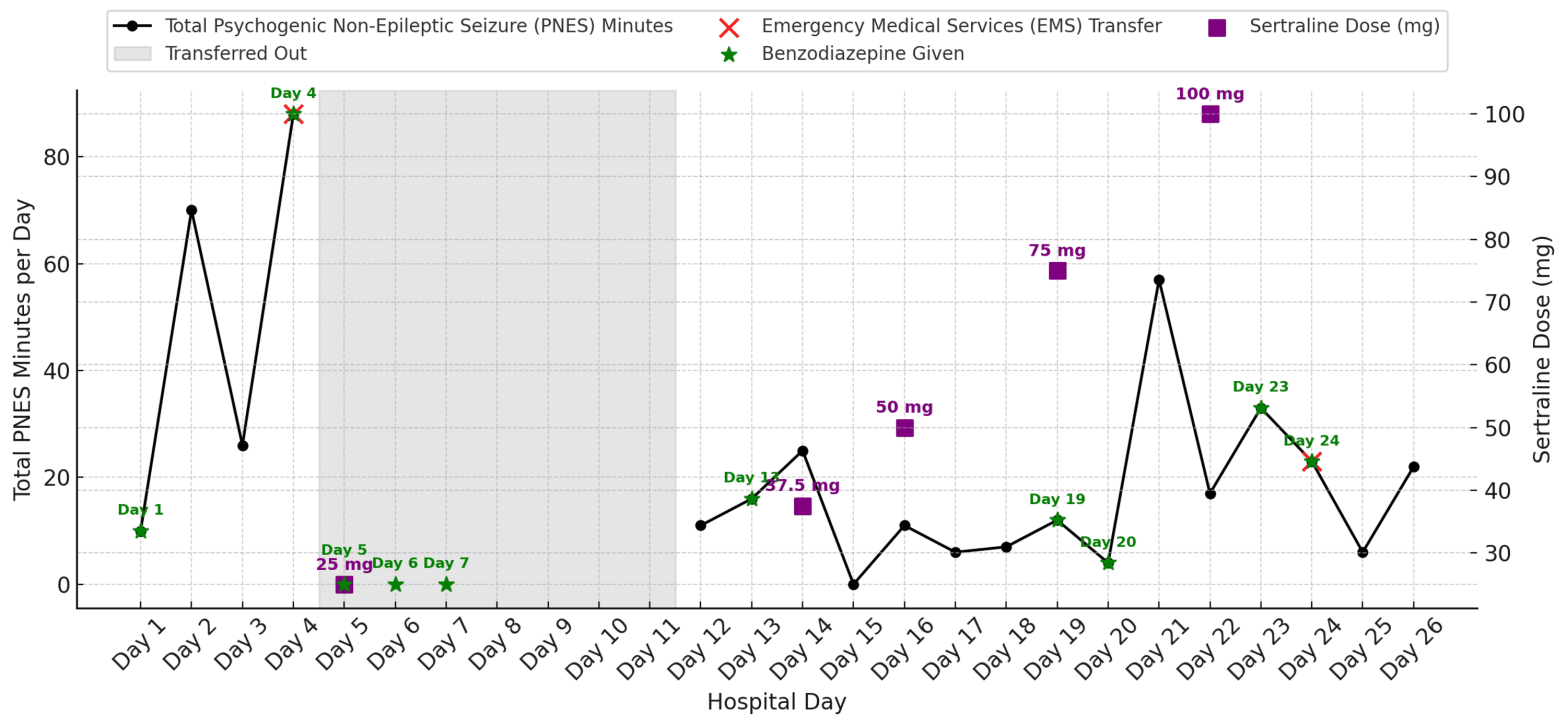
Total PNES minutes documented on each hospital day with days of sertraline titration and administration of benzodiazepine medications The figure demonstrates fluctuations in PNES burden over time in relation to medication adjustments. The black line shows total minutes of PNES activity on each hospital day. EMS transfers for acute medical evaluation are marked by a red “X”. Purple squares represent sertraline once daily dosage titration points. Green stars indicate days when benzodiazepine medication (either lorazepam or diazepam) was administered. PNES times when transferred out were not included due to insufficient documentation. PNES: psychogenic non-epileptic seizure, EMS: emergency medical services Image Credit: Authors using Python (Matplotlib, Pandas; Python Software Foundation, Wilmington, DE, USA)

Treatment included a structured behavioral plan emphasizing safety, minimal reinforcement of PNES episodes, brief reassurance (“You are safe”), and redirection to routine activities after stabilization. Benzodiazepines (diazepam 2 mg orally or intramuscularly as clinically indicated) were administered sparingly as needed, not as treatment for PNES itself but for harm reduction during prolonged or high-intensity episodes. Other pharmacologic management included gradual titration of sertraline from 25 mg to 100 mg once daily (Figure 3), hydroxyzine 25 mg every six hours as needed for anxiety, and 9 mg of melatonin nightly for insomnia. She participated in daily individual and group psychotherapy incorporating mindfulness, grounding, and distress tolerance skills, and was maintained on close observation during high-risk periods. Daily PNES duration and medication adjustments are illustrated in Figure 3.

At discharge, depressive and anxiety symptoms had improved, and self-harm behaviors had decreased. She denied SI and exhibited reactive affect, future orientation, and motivation to participate in treatment. Although PNES episodes persisted throughout admission, frequency and severity had decreased, and she demonstrated greater insight into triggers and coping strategies. Discharge medications included sertraline 100 mg once daily, hydroxyzine 25 mg every six hours as needed, and melatonin 9 mg nightly. Caregiver education emphasized continuation of the behavioral and safety plans for PNES, self-harm thoughts, and SI.

Shortly after discharge, she was admitted to an outside hospital for increased seizure-like activity. At the time, she denied SI and was compliant with her safety plan, medications, and psychotherapy. During ED workup, an unwitnessed fall with altered mental status and pupillary dilation prompted stroke code activation; repeat neuroimaging and vEEG were normal, and findings were deemed consistent with PNES. Weeks later, she presented to our ED for an intentional overdose, evaluated as an exacerbation of MDD, and was transferred to an outside facility for inpatient psychiatric care (records unavailable). She had been continuing outpatient care as directed. Additional isolated records note medical transfer during that admission and a later ED visit for seizure-like activity, though details were limited. No further encounters have been documented as of this report, months later.

## Discussion

ANS dysfunction has been increasingly recognized across many psychiatric disorders, including post-traumatic stress disorder, MDD, and schizophrenia, with reduced HRV and altered pupillary responses emerging as potential biomarkers [12,13,16-18]. While lower baseline HRV has likewise been reported in PNES, contemporary descriptions of pupillary size changes are scarce, despite historical mentions of stress-related mydriasis in “convulsive pseudoseizures” [8-11,14,15]. Notably, these early observations predated modern vEEG standards and lacked objective characterization, underscoring pupillary dilation as a potential underrecognized sympathetic feature in current PNES literature.

ANS irregularities in PNES remain incompletely characterized, though they may reflect stress-related limbic-autonomic dysregulation [8-11]. In contrast, sympathetic activation, including bilateral mydriasis, during epileptic seizures is well established and attributed to ictal cortical and subcortical activation [7,8]. This likely further contributed to the diagnostic uncertainty in this case. Aside from autonomic findings, the patient’s events exhibited features consistent with PNES rather than epilepsy, including fluctuating responsiveness, prolonged duration, absence of tongue biting or incontinence, preserved airway reflexes, and normal vEEG during events [1-4]. Within this context, pupillary dilation was most consistent with transient autonomic arousal rather than epileptic activity.

Medication effects were considered. Selective serotonin reuptake inhibitors, including sertraline, have been associated with sustained or subacute pupillary dilation via serotonergic modulation of autonomic tone [19,20]. In this case, however, dilation was episodic, occurring exclusively during PNES events and resolving between episodes. Moreover, sertraline had not yet been restarted when dilation was first observed, again supporting event-related sympathetic activation.

This case further highlights how prominent autonomic signs in PNES may raise concern and lead to unnecessary escalation of care. However, pupillary dilation alone cannot distinguish PNES from epileptic seizures; its presence, particularly when reactive and occurring alongside characteristic PNES features, should be interpreted within the broader clinical and electrophysiologic context. Accurate recognition is especially important in a population with high healthcare utilization, as diagnostic clarity has been associated with symptom reduction and improved long-term outcomes [2-6].

To our knowledge, contemporary literature contains limited detailed descriptions of dynamic, reactive pupillary dilation during vEEG-confirmed PNES. Systematic investigation using objective pupillometry alongside HRV and cardiorespiratory measures is warranted to clarify the prevalence, mechanisms, and potential diagnostic implications of pupillary changes in PNES.

## Conclusions

PNES remains a diagnostic challenge given its clinical overlap with epileptic seizures and underrecognized autonomic manifestations. This case demonstrates that bilateral, reactive pupillary dilation may occur during vEEG-confirmed PNES and likely reflects transient sympathetic activation rather than epileptic activity. Awareness of this feature may reduce unnecessary escalation of care and encourage interpretation within the broader clinical and electrophysiologic context. Further research using objective autonomic measures such as pupillometry is needed to clarify the prevalence, mechanisms, and diagnostic implications of pupillary changes in PNES. Improved characterization of such autonomic features may enhance PNES diagnostic precision, optimize management, and reduce avoidable healthcare utilization.
